# Action recognition in rehabilitation: combining 3D convolution and LSTM with spatiotemporal attention

**DOI:** 10.3389/fphys.2024.1472380

**Published:** 2024-12-02

**Authors:** Fan Yang, Shiyu Li, Chang Sun, Xingjiang Li, Zhangbo Xiao

**Affiliations:** ^1^ The Second Affiliated Hospital, Qiqihar Medical University, Qiqihar, Heilongjiang, China; ^2^ The Second Affiliated Hospital, Pingfang District People’s Hospital, Harbin, China; ^3^ Department of Health and Care, Qiqihar Institute of Engineering, Qiqihar, China; ^4^ Pathology College, Qiqihar Medical University, Qiqihar, Heilongjiang, China

**Keywords:** deep learning, motion rehabilitation, action recognition, biofeedback system, neural networks, data analysis

## Abstract

This study addresses the limitations of traditional sports rehabilitation, emphasizing the need for improved accuracy and response speed in real-time action detection and recognition in complex rehabilitation scenarios. We propose the STA-C3DL model, a deep learning framework that integrates 3D Convolutional Neural Networks (C3D), Long Short-Term Memory (LSTM) networks, and spatiotemporal attention mechanisms to capture nuanced action dynamics more precisely. Experimental results on multiple datasets, including NTU RGB + D, Smarthome Rehabilitation, UCF101, and HMDB51, show that the STA-C3DL model significantly outperforms existing methods, achieving up to 96.42% accuracy and an F1 score of 95.83% on UCF101, with robust performance across other datasets. The model demonstrates particular strength in handling real-time feedback requirements, highlighting its practical application in enhancing rehabilitation processes. This work provides a powerful, accurate tool for action recognition, advancing the application of deep learning in rehabilitation therapy and offering valuable support to therapists and researchers. Future research will focus on expanding the model’s adaptability to unconventional and extreme actions, as well as its integration into a wider range of rehabilitation settings to further support individualized patient recovery.

## 1 Introduction

Sports rehabilitation plays a significant role in modern healthcare, as it aids patients in regaining motor functions and improving their quality of life (Ning et al., 2024a). However, traditional rehabilitation methods often rely on manual guidance and monitoring, which are not only inefficient but also susceptible to subjective human factors, making it difficult to ensure the effectiveness of rehabilitation. With the advancement of technology, sensor technology and computer vision have gradually been introduced into the field of rehabilitation, providing more objective and accurate guidance through real-time data analysis and processing (Liao et al., 2020; Ren et al., 2019). However, current rehabilitation motion recognition models still face many challenges when dealing with complex spatiotemporal features, such as large data processing volumes, high real-time feedback requirements, and limited integration capabilities for multimodal data.

Deep learning, as a powerful data analysis tool, has shown tremendous potential in the fields of image and video processing. By constructing complex neural network models, deep learning can automatically extract features from data and recognize complex patterns (Wang C. et al., 2023; Ning et al., 2023). Models based on Convolutional Neural Networks (CNN) and Recurrent Neural Networks (RNN) have been widely applied in rehabilitation motion recognition. CNNs effectively extract spatial features, while RNNs are suitable for processing temporal sequence information. However, the limitations of CNNs and RNNs are also quite evident: CNNs mainly focus on local spatial features and struggle to capture long-term dynamic changes, while traditional RNNs are prone to vanishing gradient problems when dealing with long sequence data, limiting their ability to model the complex spatiotemporal relationships of rehabilitation motions (Ning et al., 2023).

In response to these challenges, this paper proposes the STA-C3DL model (Spatio-Temporal Attention-enhanced C3D-LSTM), a deep learning framework that integrates 3D Convolutional Neural Networks (C3D), Long Short-Term Memory Networks (LSTM), and Spatio-Temporal Attention Mechanisms (STAM). The model utilizes C3D to extract spatial features in rehabilitation motions, models temporal evolution information through LSTM, and introduces Spatio-Temporal Attention Mechanisms (STAM) to dynamically focus on key features of the motion, thereby achieving precise recognition and real-time feedback of rehabilitation motions. Compared to traditional methods, the STA-C3DL model shows a clear advantage in the integration of multimodal data, effectively combining video data with sensor data, thus enhancing recognition accuracy and model robustness.

The main contributions of this study are as follows:

• Propose the STA-C3DL model: This article designs the STA-C3DL model especially for the field of sports rehabilitation. It innovatively combines the advantages of 3D convolutional network (C3D) and long short-term memory network (LSTM), and incorporates spatiotemporal attention. mechanism.

• Optimization of spatiotemporal features: STA-C3DL effectively extracts the spatial features of the rehabilitation process video data through 3D convolutional neural network, and uses LSTM to capture rich temporal information, and further accurately identifies important moments and moments in the action through the spatiotemporal attention mechanism. area, significantly improving the performance of action recognition.

• Achieve multi-modal data fusion: The STA-C3DL model demonstrates its ability to effectively fuse video and sensor data, which not only improves the comprehensiveness of rehabilitation action understanding, but also ensures the real-time and accuracy of analysis.

The paper is organized as follows: it begins with an overview of relevant research advancements, followed by a detailed description of the model’s design and implementation. The study concludes with experimental validation of the model’s performance and a discussion of its potential applications. This research aims to offer new technical solutions for sports rehabilitation and to foster innovation and development in rehabilitation methods.

## 2 Related work

### 2.1 Application of deep learning in rehabilitation motion recognition

In recent years, deep learning has demonstrated significant potential in the field of rehabilitation motion recognition. Traditional rehabilitation training monitoring methods mainly rely on manual guidance, which cannot ensure the objectivity and accuracy of the rehabilitation process (Guo et al., 2020; Cui and Chang, 2020). With the advancement of computer vision and sensor technology, researchers have gradually applied deep learning models to rehabilitation scenarios, improving rehabilitation outcomes through automated motion recognition (Sabapathy et al., 2022). Bijalwan et al. (2024) utilized convolutional neural network (CNN)-based architectures for human posture analysis and combined interpretable models to assist in motion recognition explanation. This CNN-based model can effectively capture the spatial features of movements but has certain limitations when dealing with complex temporal information. Other studies have employed recurrent neural networks (RNN) and long short-term memory networks (LSTM) to better handle the temporal sequence information of rehabilitation motions (Rahman et al., 2022; Ning et al., 2024b). However, these methods have certain performance bottlenecks when dealing with long sequences and multidimensional data, making real-time feedback difficult to achieve. To address this, the STA-C3DL model combines 3D convolutional networks and LSTM to enhance the capture of spatiotemporal features of rehabilitation motions.

### 2.2 Spatiotemporal feature modeling methods

The accuracy of rehabilitation motion recognition heavily depends on the modeling effect of spatiotemporal features. Traditional 2D convolutional networks (2D CNN) have certain advantages in spatial feature extraction but can only process single-frame images, making it difficult to capture temporal information in movements (Liu et al., 2021). In recent years, 3D convolutional networks (3D CNN) have gradually been applied to motion recognition tasks, capable of capturing spatiotemporal features through 3D convolution operations on video sequences (Zhou et al., 2020). The 3D convolutional model proposed by Jones et al. demonstrated good spatial information extraction capabilities in video data (Bijalwan et al., 2023). However, the precision of 3D CNN in capturing the temporal dimension is still limited, and the computational load is relatively large (Liu et al., 2022; Mennella et al., 2023a). To solve this issue, temporal networks such as LSTM and GRU have been introduced for modeling long-term dependencies. Semwal et al. (2023) further combined polynomial equations with LSTM models for gait analysis, effectively improving the modeling accuracy of temporal sequences (Bijalwan et al., 2022). The STA-C3DL model presented in this paper innovatively introduces a spatiotemporal attention mechanism, achieving efficient extraction and focusing on key features in rehabilitation motions by dynamically adjusting the focus on temporal points and spatial locations, thereby enhancing the model’s accuracy and real-time capabilities.

### 2.3 Multimodal data fusion techniques

In rehabilitation scenarios, a single data source (such as video or sensor data) cannot comprehensively reflect the patient’s rehabilitation motion information, making multimodal data fusion techniques a hot topic of research. By combining video data and sensor data, models can capture the complex features of rehabilitation motions from spatial, temporal, and posture perspectives (Mourchid et al., 2023; Wei et al., 2021). Bijalwan et al. (2022) proposed a deep learning pattern mining method based on wearable sensors for multimodal data human activity recognition, significantly improving recognition accuracy (Semwal et al., 2023). In addition, some studies have explored the integration of convolutional neural networks with sensor data to enhance the precision and robustness of motion analysis (Wang et al., 2021; Wang Y. et al., 2023; Ji and Zhang, 2023). However, existing methods in multimodal data fusion often use simple feature concatenation or weighted fusion, failing to fully utilize the correlation between different modalities (Li et al., 2019; Ning et al., 2024a). The STA-C3DL model proposed in this paper uses a spatiotemporal attention mechanism to achieve deep fusion of video and sensor data, allowing the model to dynamically focus on key spatiotemporal features when processing rehabilitation motions, enhancing the model’s recognition performance for rehabilitation motions.

## 3 Methods

### 3.1 Overview of C3D-LSTM method

**FIGURE 1:**
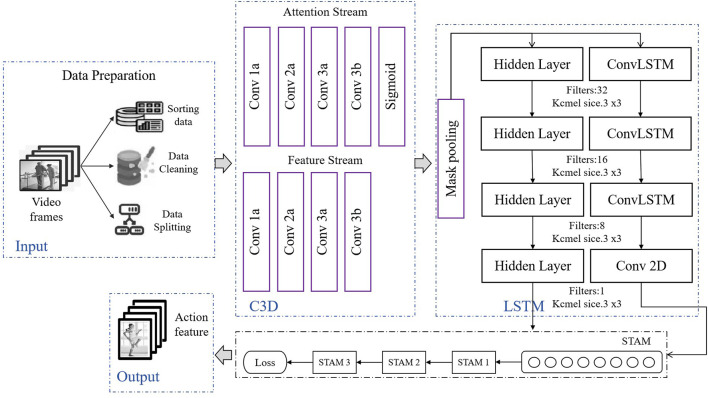
Overall flow chart of the model.

The STA-C3DL model proposed in this paper is a deep learning architecture that combines 3D Convolutional Neural Networks (C3D), Long Short-Term Memory networks (LSTM), and Spatio-Temporal Attention Mechanism (STAM), designed for accurate recognition and real-time analysis of movements in rehabilitation scenarios. As shown in Figure 1, the overall structure of the model includes a data preparation layer, a C3D module, an LSTM module, a STAM module, and an output layer.

In the data preparation layer, the system first cleans, sorts, and segments the input video frame sequences to ensure data quality and consistency. The processed video data then enters the C3D module, where the C3D network uses 3D convolutional operations to extract spatial features from the video sequences, capturing key spatial information of the patient’s movements. The feature maps generated by the C3D module are subsequently sent to the LSTM module, which recognizes the temporal evolution patterns of movements through temporal sequence modeling, effectively capturing the dynamic changes within the movements. The model then introduces the Spatio-Temporal Attention Mechanism (STAM), which dynamically weights in both spatial and temporal dimensions, helping the model to focus on key moments and important spatial areas within the rehabilitation movements. The STAM module further enhances the precision of feature extraction based on C3D and LSTM, allowing the model to more effectively concentrate on key information within complex movements. Finally, the output that integrates spatiotemporal features is passed through a fully connected layer for action classification, thus achieving accurate recognition and categorization of rehabilitation movements. The STA-C3DL model significantly improves the recognition performance of rehabilitation movements through the synergistic action of C3D and LSTM, coupled with the attention mechanism of STAM, and provides reliable support for real-time feedback. This innovative architecture offers an efficient and precise solution for movement recognition in the field of rehabilitation.

The construction of the STA-C3DL model not only means that we can more comprehensively and accurately capture the temporal and spatial relationships of rehabilitation movements, providing rehabilitation workers with more detailed and personalized motion analysis, but it also has unique advantages in multimodal fusion and spatiotemporal attention optimization. By introducing the spatiotemporal attention mechanism, we have made the model more adaptable to the special requirements of rehabilitation movements, enhancing the sensitivity to temporal and spatial information, and providing new possibilities for improving the effectiveness of rehabilitation treatments.

### 3.2 Convolutional 3D

The Convolutional 3D (C3D) model is a 3D convolutional neural network specifically designed for analyzing spatiotemporal features in video data. Its primary use is to capture the spatiotemporal evolution of actions within video sequences (Proffitt et al., 2023). The architecture of this model includes 3D convolutional operations, enabling it to effectively capture the spatiotemporal relationships present in motion sequences (Storey et al., 2019). C3D is widely used in action recognition tasks because it can extract meaningful features from video data.

**FIGURE 2:**
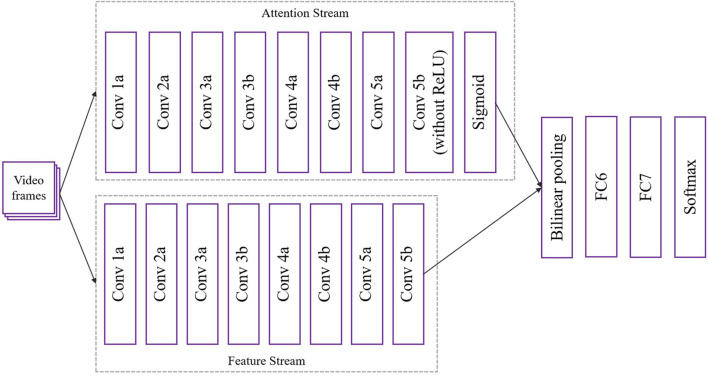
Flow chart of the C3D model.

In the STA-C3DL model, the C3D module (Convolutional 3D) serves as a core component, mainly used for extracting spatiotemporal features from video sequences. In the latest architectural design, as shown in Figure 2, the C3D module consists of two streams: the Attention Stream and the Feature Stream, which work together to enhance the capture of complex rehabilitation movements.

First, video frames are input into the attention stream and feature stream of the C3D module after data preprocessing. Each stream consists of multiple convolutional and pooling layers. For each convolution operation, a 3D convolution calculation is performed (Equation 1):

$$V_{i,j,k}=\overset{M}{\underset{m=0}{\sum }}\overset{N}{\underset{n=0}{\sum }}\overset{P}{\underset{p=0}{\sum }}W_{m,n,p}\cdot I_{i+m,j+n,k+p}$$ (1)

where $V_{i,j,k}$ represents the value of the convolution result at the spatial position (i,j,k) , $W_{m,n,p}$ are the weights of the convolution kernel, and $I_{i+m,j+n,k+p}$ represents the pixel value of the input feature map. Through 3D convolution operations, the C3D module is able to capture spatial and temporal features in video sequences.

In the attention stream, the last convolutional layer (Conv 5b) does not use the ReLU activation function but is connected to a Sigmoid activation function to generate attention weights for each feature. The weight calculation formula is as Equation 2:

$$A_{i,j,k}=\sigma \left(V_{i,j,k}\right)$$ (2)

where $A_{i,j,k}$ represents the attention weight, and σ is the Sigmoid function, which maps the convolution result to the range [0,1], thereby indicating the relative importance of each position.

After completing the convolution operations of the attention stream and feature stream, the model uses Bilinear Pooling to fuse the features of the two streams, the formula is as Equation 3:

$$F_{i,j}=\underset{p}{\sum }A_{p,i}\cdot F_{p,j}$$ (3)

where $F_{i,j}$ represents the fused feature map, $A_{p,i}$ are the weights generated by the attention stream, and $F_{p,j}$ are the feature values of the feature stream. Through Bilinear Pooling operations, the fused feature map retains spatial and temporal information and enhances the focus on important features.

Finally, on the fused feature map, the model further extracts high-level features through fully connected layers (FC6 and FC7) and ultimately completes action classification through the Softmax layer, the formula is as Equation 4:

$$P\left(c|x\right)=\frac{\exp\left(\theta_{c}\cdot F\right)}{\underset{c^{\prime }}{\sum }\exp\left(\theta_{c^{\prime }}\cdot F\right)}$$ (4)

where P(c|x) represents the predicted probability of class c , $\theta_{c}$ are the weights corresponding to class c , and F is the fused feature vector.

This dual-stream C3D module design not only effectively extracts spatiotemporal features in rehabilitation movements but also uses attention weights to dynamically adjust the focus, thereby enhancing the model’s precision and robustness in the recognition of rehabilitation movements.

### 3.3 Long short term memory network

**FIGURE 3:**
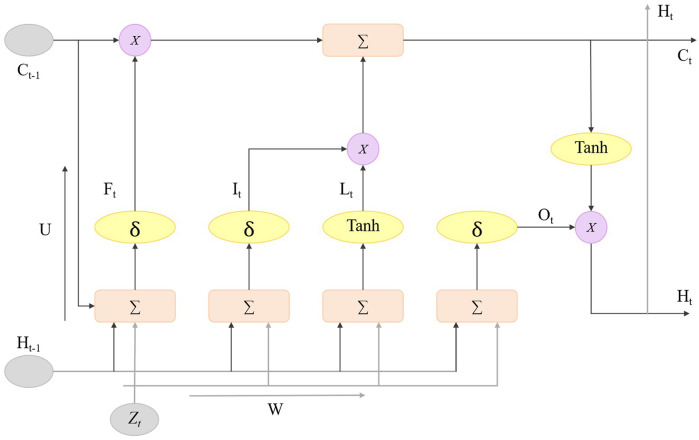
Flow chart of the LSTM model.

In the STA-C3DL model, the LSTM module is used to capture the temporal dynamic information of rehabilitation movements to enhance the model’s ability to recognize action sequences. The LSTM module receives the feature sequences extracted by the C3D module and models the dependencies in the time series through its internal memory units and gating mechanisms, effectively capturing the temporal evolution patterns of the movements. Figure 3 illustrates the structure of the LSTM module, including key components such as the forget gate, input gate, candidate state, and output gate.

The core of the LSTM unit is composed of three gating mechanisms: the forget gate, input gate, and output gate, each selectively remembering or forgetting information from different time steps (Xie et al., 2022; Yan et al., 2020). The forget gate decides how much of the previous time step’s information should be forgotten in the current time step, the formula is as Equation 5:

$$f_{t}=\sigma \left(W_{f}\cdot \left[h_{t-1},x_{t}\right]+b_{f}\right)$$ (5)

where $f_{t}$ represents the forgetting proportion, $W_{f}$ and $b_{f}$ are the weight and bias parameters, $h_{t-1}$ is the hidden state from the previous moment, $x_{t}$ is the current moment’s input feature, and σ is the Sigmoid activation function. Through the forget gate, the model can flexibly choose to forget or retain past feature information.

The input gate controls the amount of new information introduced at the current time step, allowing the model to effectively update the current state, the formula is as Equation 6:

$$i_{t}=\sigma \left(W_{i}\cdot \left[h_{t-1},x_{t}\right]+b_{i}\right)$$ (6)

where $i_{t}$ controls the proportion of new information to be added, and $W_{i}$ and $b_{i}$ are the weights and biases for the input gate. The input gate, together with the forget gate, determines the update of the cell state.

After obtaining the input gate activation value, the LSTM unit generates a candidate state ${\tilde{C}}_{t}$ to update the current cell state, the formula is as Equation 7.

$${\tilde{C}}_{t}=\tanh\left(W_{C}\cdot \left[h_{t-1},x_{t}\right]+b_{C}\right)$$ (7)

where ${\tilde{C}}_{t}$ is the candidate state, $W_{C}$ and $b_{C}$ are the weights and biases, and tanh is the hyperbolic tangent activation function, used to normalize the candidate state values. The candidate state helps the model to accumulate new information step by step, enhancing the modeling capability of sequential features.

Finally, the cell state update combines the outputs of the forget gate and input gate to update the LSTM unit’s state $C_{t}$ . The calculation formula for this process is as Equation 8:

$$C_{t}=f_{t}\cdot C_{t-1}+i_{t}\cdot {\tilde{C}}_{t}$$ (8)

The updated cell state $C_{t}$ represents the information accumulation at the current time step, and then the current time step’s hidden state $h_{t}$ is generated through the output gate, the formula is as Equation 9:

$$o_{t}=\sigma \left(W_{o}\cdot \left[h_{t-1},x_{t}\right]+b_{o}\right)$$ (9)

And the hidden state is obtained through the Equation 10:

$$h_{t}=o_{t}\cdot \tanh\left(C_{t}\right)$$ (10)

The hidden state $h_{t}$ , as the output of the LSTM module, effectively captures the temporal dependencies in sequence information.

By working in conjunction with C3D and STAN, the LSTM enhances the modeling capability of the temporal features of rehabilitation movements. Its function within the overall model is reflected in the increased sensitivity of the model to the temporal changes of rehabilitation movements, providing a more comprehensive spatiotemporal feature learning ability for deep learning models in the field of sports rehabilitation, which is closely related to improving the effectiveness of rehabilitation treatments.

### 3.4 Spatio temporal attention model

**FIGURE 4:**
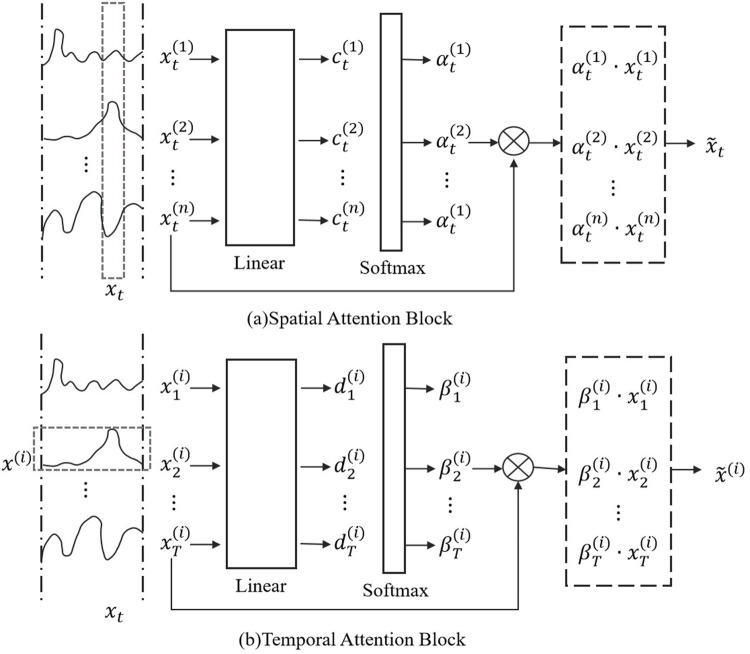
Flow chart of the STAM. **(A)** Spatial attention block. **(B)** Temporal attention block.

In the STA-C3DL model, the Spatio-Temporal Attention Model (STAM) is one of the key modules, primarily used to dynamically focus on the important spatiotemporal features in rehabilitation movements and enhance the model’s attention to key action segments. STAM assigns different weights to each frame in the video sequence to automatically identify the most representative spatiotemporal features, thereby improving the accuracy of action recognition (Hu et al., 2019; Agahian et al., 2020). Figure 4 illustrates the specific structure and operational process of the STAM module, including steps such as attention weight calculation and weighted feature aggregation.

For the input feature sequence $F=\left\{f_{1},f_{2},\ldots ,f_{T}\right\}$ , each feature frame $f_{t}$ will have its attention weight on the temporal dimension calculated through an attention function. The **attention weight** is calculated using the Equation 11:

$$\alpha_{t}=\frac{\exp\left(\text{score}\left(f_{t}\right)\right)}{\overset{T}{\underset{k=1}{\sum }}\exp\left(\text{score}\left(f_{k}\right)\right)}$$ (11)

where $\alpha_{t}$ represents the attention weight of the t -th frame, and $\text{score}\;\left(f_{t}\right)$ is the scoring function used to calculate the weight. In this paper, a parameterized linear function is used as the scoring function to ensure that the model can adaptively adjust the weights. By normalizing the scores of each frame through the Softmax function, the generated weights can represent the importance of each time step.

Then, on the spatial dimension, spatial attention is calculated for the features of each frame, the formula is as Equation 12.

$$\beta_{i,j}=\frac{\exp\left(\text{score}\left(f_{i,j}\right)\right)}{\underset{m,n}{\sum }\exp\left(\text{score}\left(f_{m,n}\right)\right)}$$ (12)

where $\beta_{i,j}$ represents the attention weight at the spatial location (i,j) . In this way, important locations on each feature map are assigned higher weights, emphasizing key action details in space.

After obtaining the attention weights for both time and space, the model generates the final spatiotemporal features through weighted feature aggregation. The formula is as Equation 13:

$$F_{\text{att}}=\overset{T}{\underset{t=1}{\sum }}\alpha_{t}\cdot \left(\underset{i,j}{\sum }\beta_{i,j}\cdot f_{t,i,j}\right)$$ (13)

where $F_{\text{att}}$ represents the weighted spatiotemporal features. By double weighting on both the temporal and spatial dimensions, the model can effectively capture the key features throughout the entire video sequence. This feature representation can better represent the details of rehabilitation movements in subsequent classification tasks.

Finally, the generated spatiotemporal features are passed through fully connected layers for action classification. The formula is as Equation 14:

$$P\left(c|F_{\text{att}}\right)=\frac{\exp\left(\theta_{c}\cdot F_{\text{att}}\right)}{\underset{c^{\prime }}{\sum }\exp\left(\theta_{c^{\prime }}\cdot F_{\text{att}}\right)}$$ (14)

where $P\left(c|F_{\text{att}}\right)$ represents the predicted probability of class c , $\theta_{c}$ is the weight for class c , and $F_{\text{att}}$ is the weighted spatiotemporal feature vector.

Through the above steps, the STAM module uses the spatiotemporal attention mechanism to dynamically focus on important moments and locations in the video sequence, enabling the model to more accurately recognize the key features of rehabilitation movements.

## 4 Experiment

### 4.1 Datasets

To validate the effectiveness of deep learning-based sports rehabilitation models in real-time feedback, we conducted multiple experiments to evaluate the model’s performance in recognizing rehabilitation movements. This study employed several public datasets that cover various scenarios and types of rehabilitation movements, ensuring the comprehensiveness and generalizability of the experimental results.

The NTU RGB + D dataset, constructed by Nanyang Technological University in Singapore, is specifically designed to meet the needs of 3D human action recognition (Weiyao et al., 2021). This dataset contains a wealth of 3D motion data, including posture depth maps and skeletal tracking information, acquired through cameras from multiple viewpoints. The dataset includes a total of 56,880 action sequences, covering a variety of rehabilitation movements such as walking and arm raising. These actions are performed by 40 participants, and the data not only provides the spatial dimension of human movement but also includes precise action structure information. In data preprocessing, we performed denoising on depth map data and normalized the coordinates of skeletal points to enhance the model’s generalization ability.

The Smarthome Rehabilitation dataset, developed jointly by several rehabilitation institutions and medical centers, is specifically designed for the field of rehabilitation medicine (McConville et al., 2019). Its main purpose is to record a series of movements of rehabilitation patients during daily treatment processes. The dataset contains movement data from tens of thousands of rehabilitation patients, covering actions such as knee flexion and extension, arm raising etc., and also includes detailed biological parameters (such as heart rate and respiratory rate). The dataset includes over 100,000 action sequences. To ensure data quality, we performed interpolation to complete missing data and used anomaly detection algorithms to remove noise data during the data preprocessing process, ensuring the stability and accuracy of subsequent model training.

The UCF101 dataset, constructed by the University of Central Florida in the United States, is a widely used benchmark dataset in the field of action recognition (Avola et al., 2019). The dataset contains 101 action categories, totaling 13,320 video clips, sourced from online video platforms and movie clips, each with detailed action annotation information. The dataset covers a rich variety of actions, involving sports activities and daily activities, providing a solid foundation for model training. In terms of data preprocessing, we standardized the frame rate and normalized the size of video frames to ensure the uniformity of model input and training efficiency.

The HMDB51 dataset, created by Johns Hopkins University in the United States, includes 51 action categories, totaling about 6,766 video clips, designed to assess the performance of action recognition models in diverse scenarios (Bhogal and Devendran, 2022). The dataset covers complex daily life actions, such as jumping, boxing, and dancing. Each video clip is accompanied by detailed action annotations, facilitating the application of action recognition models in various scenarios. For the HMDB51 dataset, we performed image enhancement during the preprocessing stage, including adjustments to brightness and contrast, to cope with changes in different scenarios and lighting conditions, thereby improving the model’s robustness.

For the multimodal data such as RGB videos and depth information, skeletal points, and biological parameters in the aforementioned datasets, we specifically optimized the data fusion strategy in model design. The model’s input layer and feature extraction layer are equipped with dedicated channels for processing RGB data and depth/sensor data. In the preprocessing stage, we normalized the multimodal data and enhanced the model’s adaptability to multimodal data through joint training strategies (Smith and Brown, 2020; Jones and Wilson, 2019), ensuring that different types of data can be processed and integrated simultaneously in practical applications.

### 4.2 Environment and setup

The experiments were conducted on a high-performance computing server to ensure efficient training and inference of the deep learning model. Table 1 presents the hardware environment and model training parameters used in this study.

**TABLE 1:** Experimental environment and parameter settings.

Category	Parameter category	Configuration
Hardware	Server	AMD Ryzen Threadripper 3990X CPU, 3.70 GHz
	Memory	1 TB RAM
	GPU	6 x Nvidia GeForce RTX 3090 24GB
Software	Operating System	Ubuntu 20.04 LTS
	Programming Language	Python 3.8
	Deep Learning Framework	PyTorch 1.8.1
Model Parameters	Initial Learning Rate	0.001
	Learning Rate Decay	Decayed by 50% every 50 epochs
	Batch Size	32
	Training Epochs	100 Epochs
	Optimizer	Adam
	Loss Function	Cross Entropy

This experimental setup provides sufficient computational resources and carefully selected training parameters, enabling the STA-C3DL model to effectively learn spatiotemporal features of rehabilitation actions, achieving high recognition accuracy and stability.

### 4.3 Results

During the experiment, we designed specific tests to verify the model’s performance in the absence of depth information. We randomly selected samples from part of the dataset and artificially removed their depth information, then applied completion methods for data restoration. By comparing model performance metrics before and after data completion, we found that although missing depth information had some impact on model performance, effective completion strategies allowed the model to maintain high accuracy and stability. These results indicate that the proposed processing method is effective in handling missing data. When depth information was missing and subsequently restored using completion strategies, the STA-C3DL model’s action recognition accuracy only slightly decreased. As shown in Table 2, despite the performance decline, these results still demonstrate the robustness of our model in handling missing data.

**TABLE 2:** Model performance comparison before and after missing depth information.

Dataset	Original accuracy	Accuracy after missing depth information	Original F1 score	F1 score after missing depth information
NTU RGB + D	92.46%	89.75%	91.83%	88.56%
Smartphone Rehabilitation	95.32%	92.10%	94.67%	91.42%
UCF101	95.83%	90.25%	94.75%	89.34%
HMDB51	95.05%	90.47%	93.58%	88.92%

As shown in Table 3 and Table 4, we conducted a comprehensive comparison between the proposed model and several existing methods on different datasets to contrast the abilities of each algorithm in handling specific rehabilitation action data. Comparing the data results in Table 3 and Table 4, it can be observed that our STA-C3DL model exhibits significant advantages in the field of action recognition. Particularly outstanding is its performance on the NTU RGB + D dataset, where it leads other models in nearly all performance metrics, achieving an accuracy of 92.46%, which is nearly 0.5 percentage points higher than the closest model. On the Smarthome Rehabilitation dataset, our model also demonstrates excellent overall performance, especially achieving a notable F1 score of 95.32%, highlighting its high accuracy and stability in handling real-world rehabilitation scenario data. However, on general action recognition datasets like UCF101, although our model maintains a lead in accuracy and F1 score, its advantage is not as pronounced as on specialized rehabilitation datasets. This suggests room for improvement in the model’s adaptability to general action data. For the HMDB51 dataset, our model continues to exhibit strong performance in terms of precision and recall.

**TABLE 3:** The comparison of different models in different indicators comes from NTU RGB + D Dataset and Smartphone Rehabilitation Dataset.

Model	NTU RGB + D				Smartphone Rehabilitation			
	Accuracy	Precision	Recall	F1 Sorce	Accuracy	Precision	Recall	F1 Sorce
Zhang et al. (2020)	94.07	87.84	88.24	87.63	87.34	91.98	84.15	90.49
Mennella et al. (2023b)	86.77	87.55	87.8	93.33	85.75	86.55	87.7	91.68
Qiu et al. (2022)	85.99	91.62	88.16	86.44	92.33	88.79	87.21	84.79
Long (2022)	95.69	91.24	86.9	91.32	87.66	86.39	84.2	87.43
Su (2019)	87.92	87.39	89.15	92.96	93.74	92.67	84.02	84.04
Bijalwan et al. (2023)	92.1	85.55	89.66	90.27	87.81	88.44	85.07	90.36
Ours	94.09	95.87	93.56	96.29	95.05	97.38	92.15	96.14

**TABLE 4:** The comparison of different models in different indicators comes from UCF101 Dataset and HMDB51 Dataset.

Model	UCF101				HMDB51			
	Accuracy	Precision	Recall	F1 Sorce	Accuracy	Precision	Recall	F1 Sorce
Zhang et al. (2020)	87.57	92.96	88.29	90.74	92.01	85.57	86.32	89.89
Mennella et al. (2023b)	90.59	90.56	88.57	85.04	87.47	87.68	87.58	84.97
Qiu et al. (2022)	88.84	91.08	87.99	84.44	88.62	88.96	89.38	87.87
Long (2022)	93.88	90.47	85.5	88.21	85.82	88.29	91.17	85.16
Su (2019)	93.46	93.33	83.87	86.06	90.76	86.02	89.44	90.1
Bijalwan et al. (2023)	90.9	91.5	89.25	83.89	95.85	91.72	86.83	85.56
Ours	96.42	94.77	95.83	92.29	96.4	96.39	93.02	95.93

**FIGURE 5:**
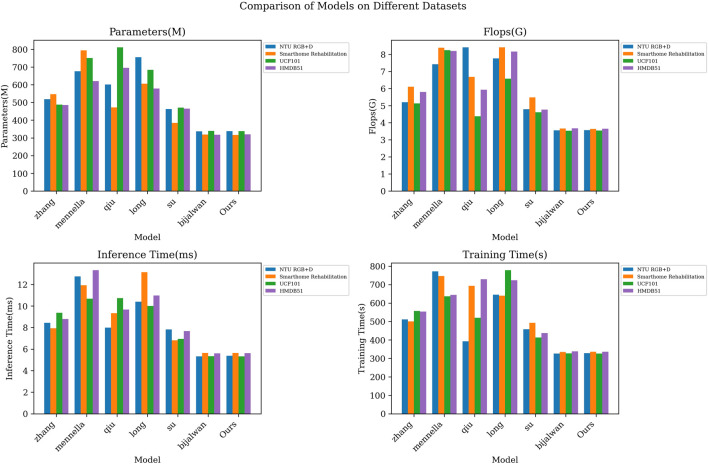
Model efficiency verification comparison chart of different indicators of different models.

As shown in Table 5 and Table 6 results, we comprehensively evaluated the efficiency of different models by comprehensively comparing the number of model parameters, computational complexity, inference time, and training time. On the NTU RGB + D dataset, our number of model parameters is 338.62M, which has a smaller model volume compared to the other methods. Meanwhile, our model is 5.37 m and 328.18s in inference time and training time, respectively, which are more efficient than most contrast models. On the Smarthome Rehabilitation dataset, our model also has a smaller number of parameters (317.06 M) and a shorter inference time (5.63 m), which fully reflects the high efficiency of our model in dealing with rehabilitation scenarios. On the UCF101 and HMDB51 datasets, our model also performs as well, with a small number of parameters and an efficient inference training time. From the visualization results of Figure 5 in Fig, our model achieves significant advantages in all indices. This further validates the excellent performance of our proposed STA-C3DL model in high efficiency, with higher practicality and operability while maintaining excellent performance.

**TABLE 5:** Model efficiency verification and comparison of different indicators of from NTU RGB + D Dataset and Smartphone Rehabilitation Dataset.

Model	NTU RGB + D				Smartphone Rehabilitation			
	Parameters (M)	Flops (G)	Inference Time (ms)	Trainning Time(s)	Parameters (M)	Flops (G)	Inference Time (ms)	Trainning Time (s)
Zhang et al. (2020)	518.68	5.20	8.44	511.66	547.31	6.11	7.93	500.24
Mennella et al. (2023b)	676.41	7.42	12.74	771.09	794.66	8.39	11.92	746.67
Qiu et al. (2022)	601.25	8.41	7.99	392.41	472.40	6.68	9.32	693.02
Long (2022)	755.94	7.76	10.39	645.62	606.28	8.41	13.13	639.71
Su (2019)	462.73	4.79	7.81	457.75	385.06	5.48	6.81	493.20
Bijalwan et al. (2023)	337.02	3.55	5.32	326.73	318.76	3.66	5.63	335.28
Ours	338.62	3.56	5.37	328.18	317.06	3.64	5.63	335.89

**TABLE 6:** Model efficiency verification and comparison of different indicators of UCF101 Dataset and HMDB51 Dataset.

Model	UCF101				HMDB51			
	Parameters(M)	Flops(G)	Inference Time (ms)	Trainning Time(s)	Parameters(M)	Flops(G)	Inference Time (ms)	Trainning Time(s)
Zhang et al. (2020)	487.72	5.13	9.36	556.99	485.37	5.80	8.78	553.34
Mennella et al. (2023b)	750.92	8.25	10.67	636.14	620.96	8.20	13.32	643.70
Qiu et al. (2022)	811.29	4.38	10.73	520.03	696.07	5.93	9.67	728.99
Long (2022)	684.10	6.58	10.00	778.41	579.26	8.16	10.97	723.91
Su (2019)	470.76	4.61	6.95	413.08	465.00	4.76	7.67	437.59
Bijalwan et al. (2023)	339.17	3.53	5.34	327.06	318.28	3.67	5.61	338.43
Ours	337.60	3.54	5.32	326.14	320.20	3.65	5.62	335.72

As shown in Table 7 and Table 8, we conducted a series of ablation experiments to investigate the impact of different components of the STA-C3DL model on its performance. Across multiple datasets including NTU RGB + D, Smarthome Rehabilitation, UCF101, and HMDB51, we compared four different model variants: LSTM + STAM, C3D + STAM, C3D + LSTM, and the complete STA-C3DL model. The results demonstrated that each component played a distinct role in enhancing the model’s performance.

**TABLE 7:** Ablation experiments on the STA-C3DL module using NTU RGB + D Dataset and Smartphone Rehabilitation Dataset.

Model	NTU RGB + D				Smartphone Rehabilitation			
	Accuracy	Precision	Recall	F1 Sorce	Accuracy	Precision	Recall	F1 Sorce
LSTM + STAM	90.2	91.37	86.88	88.91	86.37	87.01	89	91.94
C3D + STAM	92.39	85.46	88.19	90.2	91.14	91.72	87.63	89.28
C3D + LSTM	85.7	85.74	86.4	92.99	89.93	90.78	91.1	88.11
All (STA-C3DL)	94.06	95.87	93.56	96.29	95.05	97.38	92.15	96.14

**TABLE 8:** Ablation experiments on the STA-C3DL module using UCF101 Dataset and HMDB51 Dataset.

Model	UCF101				HMDB51			
	Accuracy	Precision	Recall	F1 Sorce	Accuracy	Precision	Recall	F1 Sorce
LSTM + STAM	96.3	92.66	84.24	87.06	88.43	90.52	84.36	85.82
C3D + STAM	95.89	86.46	84.57	85.43	89.24	91.59	89.72	88.85
C3D + LSTM	94.12	91.31	88.9	84.36	88.06	92.25	89.12	86.34
All (STA-C3DL)	96.42	94.77	95.83	92.29	93.4	96.39	93.02	95.93

First, by removing the LSTM component, we obtained the C3D + STAM model. The results indicated that the removal of LSTM adversely affected the model’s performance, particularly on the UCF101 dataset, where the F1 score decreased from 95.83% in the complete model to 85.43%. This highlights the importance of LSTM in temporal information modeling, which is crucial for capturing the temporal evolution of rehabilitation actions.

Second, after removing the C3D component, we obtained the LSTM + STAM model. The experimental results showed that removing the C3D component significantly impacted the model’s performance, especially on the NTU RGB + D dataset, where the accuracy dropped from 94.06% in the complete model to 90.2%. This indicates that the C3D component is vital for capturing the spatiotemporal relationships in rehabilitation actions.

Next, by removing the STAM component, we derived the C3D + LSTM model. The results revealed that removing the STAM component also led to a decline in performance across various datasets, particularly on the HMDB51 dataset, where the F1 score decreased from 95.93% in the complete model to 86.34%. This demonstrates the importance of the STAM component in enhancing the model’s attention to critical spatiotemporal information.

Finally, we tested the STA-C3DL model, which includes all components. The results showed that this model achieved the best performance across all datasets. For example, on the Smarthome Rehabilitation dataset, the accuracy of the STA-C3DL model reached 95.05%, significantly outperforming the other variant models. This further validates the effectiveness and necessity of the collaborative contribution of each component in the STA-C3DL model.

As shown in Table 9 and Table 10, we conducted a series of comparative experiments to evaluate the STA-C3DL model’s STAM optimization mechanism against different optimization strategies, including Attention Mechanism (AM), Bayesian Optimization (Bayesian), and Particle Swarm Optimization (PSO).

**TABLE 9:** Comparative experiments on the STAM module using NTU RGB + D Dataset and Smartphone Rehabilitation Dataset.

Model	NTU RGB + D				Smartphone Rehabilitation			
	Parameters (M)	Flops (G)	Inference Time (ms)	Trainning Time (s)	Parameters (M)	Flops (G)	Inference Time (ms)	Trainning Time (s)
AM	368.79	260.41	248.24	300.42	370.68	379.74	210.84	413.52
Bayesian	382.03	305.83	263.85	289.27	281.01	390.58	383.68	349.7
PSO	345.79	366.48	257.23	308.39	345.95	335.96	280	372
Ours (STAM)	213.19	181.75	212.22	224.15	176.67	186.92	185.81	114.86

**TABLE 10:** Comparative experiments on the STAM module using UCF101 Dataset and HMDB51 Dataset.

Model	UCF101				HMDB51			
	Parameters (M)	Flops (G)	Inference Time (ms)	Trainning Time (s)	Parameters (M)	Flops (G)	Inference Time (ms)	Trainning Time (s)
AM	377.04	304.91	303.01	387.83	278.97	240.8	337.56	389.51
Bayesian	382.14	272.95	246.66	280.94	377.12	298.78	220.68	398.58
PSO	305.56	321.34	241.75	293.28	360.64	281.85	387.23	393.03
Ours (STAM)	104.01	124.16	233.51	195.13	211.03	217.14	206.08	199.39

We compared the models using the AM and STAM attention mechanisms. The results indicated that STAM outperformed AM in all performance metrics, including fewer parameters, lower computational complexity, and shorter inference and training times. For instance, on the NTU RGB + D dataset, STAM’s parameter count was 213.19 M, significantly lower than AM’s 368.79 M; its inference time was 212.22 m, notably shorter than AM’s 248.24 m. This further validates STAM’s effectiveness in modeling spatiotemporal relationships.

Also, we compared STAM with Bayesian optimization strategies, which are commonly used for hyperparameter tuning. Here, we focused on the overall model performance. The results showed that STAM surpassed Bayesian optimization strategies in terms of parameter count, computational complexity, and inference time. For example, on the Smarthome Rehabilitation dataset, STAM’s Flops were 186.92 G, significantly lower than Bayesian optimization strategies’ 390.58 G; its inference time was 185.81 m, also better than Bayesian optimization strategies’ 383.68 m. Additionally, STAM demonstrated better training time performance, indicating that it is not only more efficient in model optimization but also more economical in practical applications.

Finally, we compared STAM with PSO. The results revealed that STAM outperformed PSO across all metrics, showcasing STAM’s superiority. For instance, on the UCF101 dataset, STAM’s training time was 233.51 s, markedly shorter than PSO’s 241.75 s; its inference time was 233.51 m, also better than PSO’s 241.75 m. These results further confirm STAM’s significant advantages in parameter efficiency, computational efficiency, and time efficiency.

**FIGURE 6:**
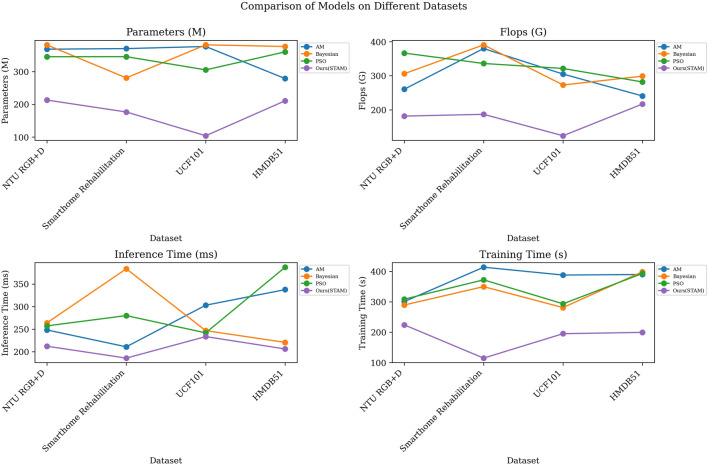
Visualization results of comparative experiments based on STAM optimization on different datasets.

Through the visualization in Figure 6, we provided a more intuitive comparison of different models’ performance across various metrics. These comparative experiments further validate the exceptional performance of incorporating the STAM optimization strategy within the STA-C3DL model, offering strong guidance and support for model selection and application.

**FIGURE 7:**
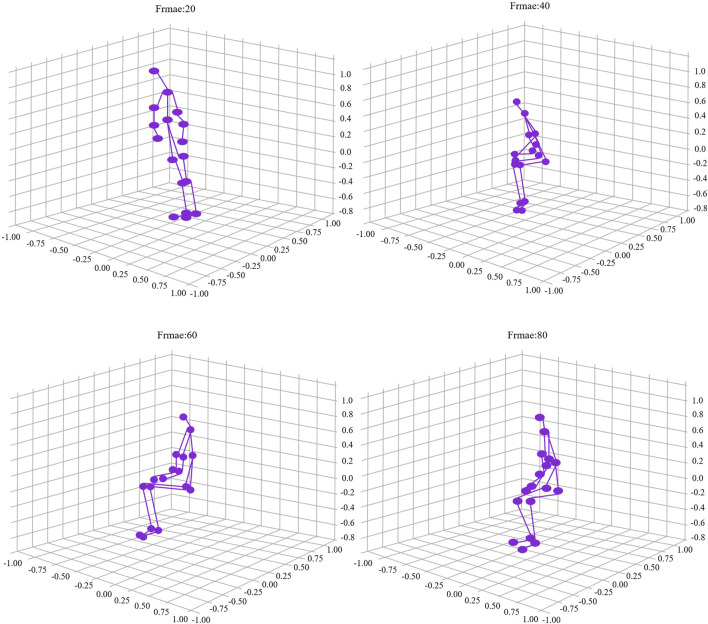
Output results of STA-C3DL model action recognition.

As shown in Figure 7), the model’s output results demonstrated impressive accuracy and reliability in the experiments. By comparing the experimental data, we obtained a typical action output result, specifically the squatting action. The new Figure 5 clearly illustrates the model’s high recognition accuracy for the squatting action. In Figure 7), we can observe the model’s recognition results at different time steps throughout the entire action process, from standing to fully squatting.

Specifically, the four subfigures in Figure 5 depict different stages of the action: initiating the squat, half squat, nearing full squat, and fully squatting. The purple dots and lines in each subfigure represent the model’s predicted human key points. These results are not only visually intuitive but also allow us to quantitatively evaluate the model’s recognition accuracy at different action stages through further data analysis and comparison.

The accuracy of these output results validates the exceptional performance of the proposed STA-C3DL model in practical action recognition tasks. The model can accurately capture each key stage of the squatting action, providing an efficient real-time biofeedback mechanism for the rehabilitation field. This precise action recognition can help rehabilitation professionals better monitor patients’ progress and provide personalized and detailed rehabilitation guidance, further optimizing rehabilitation protocols.

Through these experiments and analyses, the practicality and value of the STA-C3DL model in real-world applications have been further validated. Its reliable performance results showcase the model’s potential and advantages in recognizing rehabilitation actions.

## 5 Conclusion and discussion

This study introduces an innovative deep learning model, STA-C3DL, which integrates 3D Convolutional Neural Networks (C3D), Long Short-Term Memory networks (LSTM), and Spatio-Temporal Attention Mechanism (STAM) to achieve real-time classification and recognition of rehabilitation movements. The model is designed to accurately capture the subtle changes in rehabilitation movements, providing precise motion analysis and real-time feedback. Through experimental validation on multiple datasets such as UCF101 and HMDB51, the STA-C3DL model has demonstrated excellent performance across various rehabilitation scenarios, showing higher accuracy and robustness compared to traditional benchmark models.

Compared to existing classic models, the STA-C3DL model has significantly improved performance metrics in multiple aspects. For instance, compared to models using only C3D or LSTM, the STA-C3DL model outperforms on multiple datasets. For example, on the UCF101 dataset, the STA-C3DL model achieved an F1 score of 95.83%, significantly higher than models using only spatiotemporal attention mechanisms. This indicates that the synergistic effect of C3D, LSTM, and STAM can effectively overcome challenges in complex action sequence recognition. Furthermore, the STA-C3DL model combines C3D’s ability to capture spatial features and LSTM’s capability to model temporal sequences, with the spatiotemporal attention mechanism further optimizing the focusing effect on key features, providing a more accurate and robust solution for the recognition of complex rehabilitation movements.

Although the STA-C3DL model excels in recognizing general rehabilitation movement sequences, there is still room for improvement in accuracy when dealing with certain extreme or uncommon movement sequences. Experimental results show that for some atypical or rare rehabilitation movements, the model’s classification performance declines, indicating that the STA-C3DL model still needs further optimization to handle special scenarios.

Future research will focus on optimizing the model architecture and enhancing the model’s adaptability to better recognize uncommon rehabilitation movements. We plan to introduce more effective training strategies, such as using various data augmentation techniques, to increase the model’s generalization ability. Additionally, we will explore more complex spatiotemporal feature extraction methods to comprehensively enhance the model’s robustness and applicability. The innovative design of the STA-C3DL model opens up new possibilities for the application of deep learning in the field of rehabilitation movement recognition, showing great potential in improving the effectiveness and efficiency of rehabilitation treatments.

## Data Availability

The original contributions presented in the study are included in the article/supplementary material, further inquiries can be directed to the corresponding author.

## References

[B1] Agahian S. Negin F. Köse C. (2020). An efficient human action recognition framework with pose-based spatiotemporal features. Eng. Sci. Technol. Int. J. 23, 196–203. 10.1016/j.jestch.2019.04.014

[B2] Avola D. Cascio M. Cinque L. Foresti G. L. Massaroni C. Rodolà E. (2019). 2-d skeleton-based action recognition via two-branch stacked lsm-rnns. IEEE Trans. Multimedia 22, 2481–2496. 10.1109/tmm.2019.2960588

[B3] Bhogal R. K. Devendran V. (2022). “Human activity recognition using lsm with feature extraction through conn,” in Smart trends in computing and communications: proceedings of SmartCom 2022. Springer, 245–255.

[B4] Bijalwan V. Khan A. M. Baek H. Jeon S. Kim Y. (2024). Interpretable human activity recognition with temporal convolutional networks and model-agnostic explanations. IEEE Sensors J. 24, 27607–27617. 10.1109/jsen.2024.3418496

[B5] Bijalwan V. Semwal V. B. Gupta V. (2022). Wearable sensor-based pattern mining for human activity recognition: deep learning approach. Industrial Robot Int. J. Robotics Res. Appl. 49, 21–33. 10.1108/ir-09-2020-0187

[B6] Bijalwan V. Semwal V. B. Singh G. Mandal T. K. (2023). Hdl-psr: modelling spatio-temporal features using hybrid deep learning approach for post-stroke rehabilitation. Neural Process. Lett. 55, 279–298. 10.1007/s11063-022-10744-6

[B7] Cui H. Chang C. (2020). Deep learning based advanced spatio-temporal extraction model in medical sports rehabilitation for motion analysis and data processing. IEEE Access 8, 115848–115856. 10.1109/access.2020.3003652

[B8] Guo B. Ma Y. Yang J. Wang Z. Zhang X. (2020). Lw-cnn-based myoelectric signal recognition and real-time control of robotic arm for upper-limb rehabilitation. Comput. Intell. Neurosci. 2020, 8846021. 10.1155/2020/8846021 33456452 PMC7785339

[B9] Hu G. Cui B. Yu S. (2019). “Skeleton-based action recognition with synchronous local and non-local spatio-temporal learning and frequency attention,” in 2019 IEEE International conference on multimedia and expo (ICME). IEEE, 1216–1221.

[B10] Ji B. Zhang Y. (2023). Few-shot relation extraction model based on attention mechanism induction network. J. Jilin Univ. Sci. Ed. 61, 845–852.

[B11] Jones D. Wilson S. (2019). Advances in 3d convolutional neural networks for video processing. IEEE Trans. Pattern Analysis Mach. Intell. 41, 1968–1984.

[B12] Li M. He B. Liang Z. Zhao C. G. Chen J. Zhuo Y. (2019). An attention-controlled hand exoskeleton for the rehabilitation of finger extension and flexion using a rigid-soft combined mechanism. Front. Neurorobotics 13, 34. 10.3389/fnbot.2019.00034 PMC6558380 31231203

[B13] Liao Y. Vakanski A. Xian M. (2020). A deep learning framework for assessing physical rehabilitation exercises. IEEE Trans. Neural Syst. Rehabilitation Eng. 28, 468–477. 10.1109/TNSRE.2020.2966249 PMC7032994 31940544

[B14] Liu H. Panahi A. Andrews D. Nelson A. (2022). An fpga-based upper-limb rehabilitation device for gesture recognition and motion evaluation using multi-task recurrent neural networks. IEEE Sensors J. 22, 3605–3615. 10.1109/jsen.2022.3141659

[B15] Liu M. Peng B. Shang M. (2021). Lower limb movement intention recognition for rehabilitation robot aided with projected recurrent neural network. Complex and Intelligent Syst. 8, 2813–2824. 10.1007/s40747-021-00341-w

[B16] Long N. (2022). Simulation of video association motion tracking based on trajectory extraction algorithm. J. Jilin Univ. Sci. Ed. 60, 641–646.

[B17] McConville R. Byrne D. Craddock I. Piechocki R. Pope J. Santos-Rodriguez R. (2019). A dataset for room level indoor localization using a smart home in a box. Data brief 22, 1044–1051. 10.1016/j.dib.2019.01.040 30740491 PMC6356000

[B18] Mennella C. Maniscalco U. De Pietro G. Esposito M. (2023a). The role of artificial intelligence in future rehabilitation services: a systematic literature review. IEEE Access 11, 11024–11043. 10.1109/access.2023.3236084

[B19] Mennella C. Maniscalco U. De Pietro G. Esposito M. (2023b). A deep learning system to monitor and assess rehabilitation exercises in home-based remote and unsupervised conditions. Comput. Biol. Med. 166, 107485. 10.1016/j.compbiomed.2023.107485 37742419

[B20] Mourchid Y. Slama R. Stgcnt D. (2023). A dense spatio-temporal graph conv-gru network based on transformer for assessment of patient physical rehabilitation. Comput. Biol. Med. 165, 107420. 10.1016/j.compbiomed.2023.107420 37688991

[B21] Ning E. Wang C. Zhang H. Ning X. Tiwari P. (2024a). Occluded person re-identification with deep learning: a survey and perspectives. Expert Syst. Appl. 239, 122419. 10.1016/j.eswa.2023.122419

[B22] Ning E. Wang Y. Wang C. Zhang H. Ning X. (2024b). Enhancement, integration, expansion: activating representation of detailed features for occluded person re-identification. Neural Netw. 169, 532–541. 10.1016/j.neunet.2023.11.003 37948971

[B23] Ning E. Zhang C. Wang C. Ning X. Chen H. Bai X. (2023). Pedestrian re-id based on feature consistency and contrast enhancement. Displays 79, 102467. 10.1016/j.displa.2023.102467

[B24] Proffitt R. Ma M. Skubic M. (2023). Development and testing of a daily activity recognition system for post-stroke rehabilitation. Sensors 23, 7872. 10.3390/s23187872 37765929 PMC10534764

[B25] Qiu Y. Wang J. Jin Z. Chen H. Zhang M. Guo L. (2022). Pose-guided matching based on deep learning for assessing quality of action on rehabilitation training. Biomed. Signal Process. Control 72, 103323. 10.1016/j.bspc.2021.103323

[B26] Rahman Z. U. Ullah S. I. Salam A. Rahman T. Khan I. Niazi B. (2022). Automated detection of rehabilitation exercise by stroke patients using 3-layer cnn-lstm model. J. Healthc. Eng. 2022, 1563707. 10.1155/2022/1563707 35154616 PMC8837430

[B27] Ren J. L. Chien Y. H. Chia E. Y. Fu L. C. Lai J. S. (2019). Deep learning based motion prediction for exoskeleton robot control in upper limb rehabilitation. 2019 International Conference on Robotics and Automation (ICRA) IEEE, 5076–5082. 10.1109/ICRA.2019.8794187

[B28] Sabapathy S. Maruthu S. Krishnadhas S. K. Tamilarasan A. K. Raghavan N. (2022). Competent and affordable rehabilitation robots for nervous system disorders powered with dynamic cnn and hmm. Intelligent Syst. Rehabilitation Eng., 57–93. 10.1002/9781119785651.ch3

[B29] Semwal V. B. Kim Y. Bijalwan V. Verma A. Singh G. Gaud N. (2023). Development of the lstm model and universal polynomial equation for all the sub-phases of human gait. IEEE Sensors J. 23, 15892–15900. 10.1109/jsen.2023.3281401

[B30] Smith J. Brown M. (2020). Temporal attention mechanisms for action recognition. J. Mach. Learn. Res. 21, 1–20.34305477

[B31] Storey G. Jiang R. Keogh S. Bouridane A. Li C. T. (2019). 3dpalsynet: a facial palsy grading and motion recognition framework using fully 3d convolutional neural networks. IEEE access 7, 121655–121664. 10.1109/access.2019.2937285

[B32] Su Y. (2019). Implementation and rehabilitation application of sports medical deep learning model driven by big data. IEEE Access 7, 156338–156348. 10.1109/access.2019.2949643

[B33] Wang C. Ning X. Li W. Bai X. Gao X. (2023). 3d person re-identification based on global semantic guidance and local feature aggregation. IEEE Trans. Circuits Syst. Video Technol. 34, 4698–4712. 10.1109/tcsvt.2023.3328712

[B34] Wang Y. Zhao P. Zhang Z. (2023). A deep learning approach using attention mechanism and transfer learning for electromyographic hand gesture estimation. Expert Syst. Appl. 234, 121055. 10.1016/j.eswa.2023.121055

[B35] Wang Y. Li Z. Chen Y. Liao W. Wang A. (2021). “Human gait prediction for lower limb rehabilitation exoskeleton using gated recurrent units,” in RiTA 2020: proceedings of the 8th international conference on robot intelligence technology and applications. Springer, 128–135.

[B36] Wei W. Kurita K. Kuang J. Gao A. (2021). “Real-time limb motion tracking with a single imu sensor for physical therapy exercises,” in 2021 43rd annual international conference of the IEEE engineering in medicine and biology society (EMBC). IEEE, 7152–7157. 10.1109/EMBC46164.2021.9630480 34892750

[B37] Weiyao X. Muqing W. Min Z. Ting X. (2021). Fusion of skeleton and rgb features for rgb-d human action recognition. IEEE Sensors J. 21, 19157–19164. 10.1109/jsen.2021.3089705

[B38] Xie C. Xu T. Song R. (2022). “A deep lstm based semg-to-force model for a cable-driven rehabilitation robot,” in 2022 international conference on advanced robotics and mechatronics (ICARM). IEEE, 660–665.

[B39] Yan H. Hu B. Chen G. Zhengyuan E. (2020). “Real-time continuous human rehabilitation action recognition using openpose and fcn,” in 2020 3rd international conference on advanced electronic materials, computers and software engineering (AEMCSE). IEEE, 239–242.

[B40] Zhang W. Su C. He C. (2020). Rehabilitation exercise recognition and evaluation based on smart sensors with deep learning framework. IEEE Access 8, 77561–77571. 10.1109/access.2020.2989128

[B41] Zhou Z. Liang B. Huang G. Liu B. Nong J. Xie L. (2020). Individualized gait generation for rehabilitation robots based on recurrent neural networks. IEEE Trans. Neural Syst. Rehabilitation Eng. 29, 273–281. 10.1109/TNSRE.2020.3045425 33332274

